# Supplementary material for: Are carbon emissions trading and green financial instruments synergistic? -Comprehensive quantitative research based on content analysis
Source: PLoS One. 2024 Mar 7;19(3):e0298601. doi: 10.1371/journal.pone.0298601 (PMC10919601; doi:10.1371/journal.pone.0298601)
Supplement: S1 Data — (ZIP) [file pone.0298601.s001.zip › Data/地级市Z_F工作报告.zip!/─┌├╔╣┼/═¿┴╔╩╨/└┤╫╘╣1⁄2╓┌║┼í╛┬φ┐╦╩2╛▌═°í┐_2016.docx]

各位代表：

现在，我代表市人民政府向大会报告工作，请与《通辽市国民经济和社会发展第十三个五年规划纲要（草案）》一并审议，并请市政协委员和列席会议的同志们提出意见。

一、“十二五”时期和2015年经济社会发展回顾

过去的五年是我市发展进程中极不平凡的五年。五年来，在市委的坚强领导下，市政府团结依靠全市各族人民，认真落实中央、自治区、市委各项决策部署，深入贯彻自治区“8337”发展思路，统筹做好稳增长、促改革、调结构、惠民生、防风险各项工作，经济社会发展取得了新的成就。

——综合经济实力不断增强。预计2015年，地区生产总值完成1900亿元，比2010年增长67.9%，年均增长10.9%；一般公共预算收入完成120.5亿元，比2010年增长85.8%，年均增长13.2%；一般公共预算支出完成370亿元，比2010年增长1倍，年均增长14.9%；社会消费品零售总额实现470亿元，比2010年增长83.9%，年均增长13%；限额以上固定资产投资完成1280亿元，比2010年增长85.1%，年均增长13.1%。五年累计开工建设亿元以上项目387个，为新常态下实现新发展注入了强劲动力。

——经济结构进一步优化。三次产业结构调整为16:49:35，正在向一产稳、二产优、三产强的方向积极转变。生态高效农牧业发展格局初步形成，节水高产高效粮食功能区达到850万亩，压减农用机电井3万眼，年节水5.4亿立方米，粮食产量达到138亿斤，建成饲草料基地500万亩，牲畜存栏突破2000万头只大关。“三大产业集群”支撑作用显著增强，铝新材料、玉米生物科技、绿色农畜产品加工等优势产业生产规模和科技水平大幅提升。第三产业比重进一步提高，电子商务、现代物流、文化旅游、金融保险、特色餐饮等现代服务业增长势头强劲。非公经济成为促进经济增长、扩大社会就业的重要力量。

——城乡面貌发生新变化。全力以赴推进“十个全覆盖”工程，2014年以来，累计投资134亿元，完成1644个行政嘎查村、115个自然村、32个国有农牧场分场建设任务，农村牧区面貌焕然一新。各级干部群众用自己的辛苦指数换来了农牧民的幸福指数，广大群众为党的好政策、干部的好作风点赞。新型城镇化步伐加快，全市常住人口城镇化率达到46.5%，比2010年提高6.1个百分点。市政公用建设水平大幅提高，全市建成区绿化覆盖率等指标进入自治区前列，成功创建国家园林城市。

——基础设施保障能力得到提升。新建和改建公路里程7369公里，与北京、东北省会城市实现高速公路连接，主城区至旗县实现高等级公路连通，通辽至鲁北高速公路开工建设，行政嘎查村公路通畅率达到77%。通辽至京沈高铁连接线开工建设，通辽人的“高铁时代”即将到来。昌图至通辽天然气管道工程投入使用，开鲁至科尔沁500千伏输电线路基本完工。完成西辽河治理一期工程，毛都水库开工建设。完成林业生态建设884.3万亩，森林覆盖率提高2.68个百分点、达到28%，科尔沁沙地在全国四大沙地中率先实现良性逆转。严格环境准入和污染防治，集中整治和解决了一批环境隐患问题，环境质量总体保持稳定。

——民生保障和社会事业取得新成绩。城镇常住居民人均可支配收入预计比2010年增长75.8%、达到25481元，年均增长11.9%；农村牧区常住居民人均可支配收入预计比2010年增长96.2%、达到10925元，年均增长14.4%。累计新增城镇就业8.6万人，被国务院命名为“全国创业先进城市”。36万贫困人口实现稳定脱贫。70多万城乡居民圆了“安居梦”。城乡公共文化、全民健身、医疗卫生、社会保障和救助体系日臻完善。在全区率先实现苏木乡镇、中心嘎查村幼儿园全覆盖。民族团结进步创建活动深入开展。“六五”普法圆满结束。“平安通辽”建设成效明显。军民军地融合发展。史志、档案、气象、人防、新闻出版、防震减灾、慈善救助、外事侨务、妇女儿童、文化遗产保护、残疾人和老龄工作、关心下一代及青少年思想道德建设等各项社会事业全面进步。

2015年是令人难忘的一年。面对严峻复杂的经济形势和艰巨繁重的改革发展任务，全市各族人民在市委的坚强领导下，牢牢把握稳中求进工作总基调，主动适应经济发展新常态，与时俱进完善发展思路，凝心聚力破解发展难题，进一步开创了经济社会发展新局面。

（一）全力稳定经济增长。面对经济下行压力，我们及时制定出台了稳定工业增长50条意见、促进房地产市场健康发展40条意见、支持服务业发展5个配套文件等政策措施。全年实施500万元以上固定资产投资项目1497个，其中工业项目283个。列入自治区和市本级调度的76个重点项目全部开复工。全年发电量475亿度，增长20%；工业用电量382亿度，增长25%；协调落实大用户直供电交易量73亿度，占蒙东地区总交易电量的87.6%，为企业节约用电成本7亿元。国家发改委核准我市电解铝产能312.5万吨。通过“助保贷”平台为174户中小企业发放贷款10.3亿元。规模以上工业企业新增43户，工业增速由年初的7.6%回升至9.2%。认真贯彻落实国家粮食收购政策，及时出台秋粮收购指导意见，在粮食丰收、粮价下调的形势下，确保敞开收购，最大限度降低因粮价变动给农牧民增收带来的负面影响。通过不懈努力，全市经济运行总体平稳，实现了稳中有进、稳中有好。

（二）加快产业转型升级。农牧业产业化、标准化、信息化迈出新步伐，农畜产品综合加工转化率达到68%，综合机械化水平达到83%；牧业年度牛存栏达到302万头，实现5万头育肥牛质量安全可追溯。工业主导产业加快延伸升级，电解铝产能新增80万吨，铝精深加工产能新增87.5万吨，新增铝后加工产品42个；玉米生物科技产业新增产业链5条、产品31个；绿色农畜产品品牌影响力进一步扩大，产品销量稳定增长。现代服务业加快发展，我市被确定为“全国自驾游目的地试点城市”，旅游接待人数、旅游综合收入大幅增长；民航旅客吞吐量达到90万人次；通辽电子商务产业园被评为“第二批全国电子商务示范基地”，全市电子商务企业达到328家，农村牧区电子商务服务站达到1180个，成功承办全区农村牧区电子商务现场会。

（三）着力推进改革开放。经济生态和社会事业领域形成各类改革成果198条，一些关键领域和重点环节改革迈出重要步伐。深入推进简政放权，公布了市、旗两级权力清单，公共资源交易项目实行分级招投标，市本级行政审批事项由193项精简为61项，审批时限平均压缩64%。实行“三证合一”、“一照一码”登记制度，市场主体增长14.3%。深化电力体制改革，北部区域微型电网建设列入自治区电力体制改革试点，锦联自备电厂2×66万千瓦机组并网发电。深化国资国企改革，先后出台12个国企改革方案，霍煤集团与内蒙古矿业集团合作重组正在积极推进。深化投融资体制改革，向社会公布政府与社会资本合作项目160个，全市“四权”抵押贷款余额突破3亿元。深化农村牧区综合改革，奈曼旗土地承包经营权确权登记颁证试点、扎鲁特旗草原确权承包改革试点进入整体验收阶段，完成了舍伯图项目区农业节水综合改革和扎鲁特旗全国小型水利工程管理体制改革试点任务。深化生态文明制度改革，划定林业生态保护红线，启动罕山等四个生物多样性保护示范区建设，在扎鲁特旗开展领导干部自然资源资产离任审计试点。加快构建开放型经济新体制，积极向俄蒙开放，扩大与周边区域合作。全年新签约亿元以上招商引资项目155个，引进市外国内资金644.9亿元，实现外贸进出口总额4.3亿美元。

（四）加强城市规划建设管理。城市总体规划（2014—2030年）修编工作基本完成。主城区开工建设了新世纪大桥、建国北路、胜利北路和北外环拓宽以及给排水、供热等市政基础设施项目，推进了图书馆新馆、科技馆、12个民族文化专题博物馆等社会事业项目。投资7.8亿元实施了老旧小区改造、自来水水质提升、公共厕所修建、环境卫生整治、小街小巷硬化亮化、交通标识设置等民生城建项目，对垃圾围城、占道经营、私搭乱建、交通秩序、广告牌匾等“城市病”进行了综合整治，城市形象和功能品位得到提升。旗县“一街一路一镇一社区”改造建设工程进展顺利，奈曼旗、科左后旗、扎鲁特旗成功创建自治区园林县城。将市级城市规划监察、环境卫生、城管执法等部分管理权限下放至科尔沁区和开发区。政府采取合理回购的办法理顺了公交管理体制，主城区公共交通条件得到改善，兑现了特殊群体乘坐公交的各项优惠政策。启动“智慧城市”建设，智慧交通、智慧安监、主城区视频信息共享平台建设效果较好。

（五）切实保障和改善民生。各级财政民生支出298.5亿元，占财政总支出的80.7%，比2014年增加42亿元，重点实施了十大民生工程。实施农村牧区“十个全覆盖”工程，当年投资91.6亿元，完成全覆盖的行政嘎查村达到944个。实施精准扶贫工程，整合投入各类扶贫资金31亿元，1.9万贫困户、7.5万贫困人口实现稳定脱贫。实施百姓安居工程，开工各类保障性住房1.89万套，改造农村牧区危房4.03万户。实施食品药品安全工程，加强源头治理和全程监管，餐厨废弃物、废弃油脂处理厂启动建设，6个旗县市区的食品加工园区投入使用。实施医疗保险统筹工程，医疗保险实现市级统筹，城乡大病医疗保险实现全覆盖，城镇职工和居民医疗保险报销比例、统筹基金年度最高支付限额进一步提高，公立医院综合改革稳步推进。实施文化体育惠民工程，城乡公共文化服务设施全部免费对外开放，成功承办全国贫困地区公共文化建设工作推进会，主城区全民健身中心开工建设，部分机关企事业单位体育场馆向社会开放，足球改革取得积极进展。实施社会事业改革创新工程，启动建设通辽国家农业科技园区，科技大市场实现技术交易额3.1亿元，推介科技成果200项。教育领域综合改革不断深化，教育教学水平不断提高，扎鲁特旗通过国家义务教育均衡发展达标验收，组建了市级职业教育集团，通辽职业学院被确定为自治区级示范性高等职业院校。深化医药卫生体制改革，基层医疗卫生服务体系不断完善，新农合参保率达到97.7%，新建7所苏木乡镇卫生院和698所嘎查村标准化卫生室，京通医疗卫生合作持续深入开展，成功创建全国基层蒙中医药先进市和全国无偿献血先进市。创新社会治理，全面推行城镇社区、农村牧区“网格化”管理。实施就业创业工程，实现零就业家庭动态清零，新增城镇就业1.73万人，开发区汇智众创基地列入自治区首批众创空间试点。实施社会保障体系完善工程，低收入农牧民家庭1吨“暖心煤”、低保家庭大学生每年1万元助学金、零就业家庭1人稳定就业政策全面兑现，城乡低保、农村牧区五保供养标准和补助水平稳步增长，调增企业退休人员养老金标准，开展特殊群体和困难家庭临时救助，城乡医疗救助实现即时结算。实施安全生产责任工程，强化安全生产责任制落实，有效遏制重特大事故发生，实现生产事故、死亡人数“双下降”。

（六）不断加强政府自身建设。认真执行人大及其常委会的决议、决定，自觉接受政协民主监督。向市人大专题报告政府工作20项，与市政协开展专题协商15项，办理市人大代表建议143件、政协委员提案276件。严格遵守重大行政决策法定程序，对城市总体规划修编、重大项目建设和重点民生工程开展专家评估论证，广泛听取社会各方面意见。市旗镇三级政府聘任法律顾问实现全覆盖。推进政务公开，开通“12345”市长公开电话，及时回应群众关切。做到新官也要理旧账，保持工作连续性和政策稳定性，将政府债务纳入预算，实行规模控制和分类管理。对部分市政府投资项目进行中期评估，审计监督作用日益加强。开展“三严三实”专题教育，认真落实党风廉政建设主体责任。市政府个别组成人员因严重违纪违法受到严厉查处。

各位代表，2015年经济社会发展目标的顺利完成，为我市“十二五”规划的全面收官画上了圆满句号。五年的砥砺前行，为我们积累了许多宝贵经验。主要是：必须坚持正确的政治方向，必须坚持发展第一要务，必须坚持调结构转方式，必须坚持深化改革扩大开放，必须坚持生态保护与建设并重，必须坚持保障和改善民生，必须坚持维护社会和谐稳定大局。我们一定要倍加珍惜弥足珍贵的经验，凝聚全市各族人民的智慧和力量，从实际出发创造性地开展工作，在新的历史起点上把我市改革开放和现代化建设不断推向前进。

各位代表，“十二五”时期取得的成绩来之不易。这是自治区党委、政府和市委总揽全局、正确领导的结果，是市人大、市政协履职监督、倾力支持的结果，是社会各界辛勤努力、无私奉献的结果，是各级干部敢于担当、真抓实干的结果，是全市各族人民守望相助、团结奋斗的结果。在此，我代表市人民政府，向全市各族人民，向所有关心支持通辽发展的同志们、朋友们，表示衷心的感谢！

在看到成绩的同时，我们必须清醒地认识到，我市经济社会发展和政府工作还存在许多不足，前进道路上还有不少困难和问题。一是经济下行压力依然较大，招商引资和项目引进竞争加剧，新的经济增长点不多；二是实体经济面临不少困难，主要工业品出厂价格下降，融资和劳动力成本上升，民营经济和小微企业发展受到瓶颈制约；三是产业层次不高，结构不合理，现代服务业发展滞后，战略性新兴产业发展不足，玉米“一粮独大”的种植结构影响农牧民持续增收；四是科技创新能力不强、投入不够，人才缺乏和流失等问题较为突出；五是财政收入增速放缓和民生刚性需求增加的矛盾逐步显现，教育医疗、文化体育、社会保障、脱贫攻坚、城市建设等领域还有大量工作要做；六是创建全国文明城市和国家卫生城市工作还不够扎实，对照《全国文明城市测评体系》，一些硬性指标仍有较大差距；七是政府自身建设有待进一步加强，有些政府组成部门还存在不敢担当、不理旧账、为官不为问题；八是一些领域违规违纪现象时有发生，反腐倡廉仍须常抓不懈、警钟常鸣，法治政府建设任务紧迫而繁重。问题所在就是努力的方向，人民期待就是工作的目标。我们将直面问题，不以事艰而退避，不以任重而不为，采取更有力措施，切实加以解决，让全市各族人民看到实实在在的成效。

二、“十三五”时期经济社会发展的主要任务

根据市委《关于制定国民经济和社会发展第十三个五年规划的建议》精神，“十三五”时期，我市经济社会发展的指导思想是：高举中国特色社会主义伟大旗帜，全面贯彻党的十八大和十八届三中、四中、五中全会精神，以马克思列宁主义、毛泽东思想、邓小平理论、“三个代表”重要思想、科学发展观为指导，深入贯彻习近平总书记系列重要讲话和考察内蒙古重要讲话精神，坚持“四个全面”战略布局，坚持发展第一要务，坚持创新、协调、绿色、开放、共享发展理念，加快形成引领经济发展新常态的体制机制和发展方式，进一步提高发展质量和效益，统筹推进经济建设、政治建设、文化建设、社会建设、生态文明建设，实现在蒙东地区率先发展，确保如期全面建成小康社会。总体目标是：到2020年全面建成小康社会，经济社会发展达到更高水平。

——经济保持中高速增长。主要经济指标增速高于自治区平均水平，提前实现地区生产总值和城乡常住居民收入比2010年翻一番的奋斗目标。

——转变发展方式取得新突破。产业发展向中高端迈进，三次产业融合发展水平不断提高。服务业增加值占地区生产总值比重达到40%以上。区域发展协调性不断增强，户籍人口城镇化率达到45%。

——多元发展、多极支撑的现代产业体系基本形成。基本建成有色金属、清洁能源、玉米生物科技、绿色农畜产品、蒙医蒙药和自驾旅游体验六大基地。

——人民生活水平和质量普遍提高。就业比较充分，城乡常住居民收入达到自治区平均水平。公共服务体系更加健全。贫困人口全部脱贫，贫困旗县全部摘帽。

——居民素质和社会文明程度显著提高。人民思想道德素质、科学文化素质、健康素质明显提高，全社会法治意识不断增强。科尔沁文化影响力不断扩大，创建全国文明城市和国家卫生城市。

——生态环境质量持续改善。主要生态系统步入良性循环，主要污染物排放总量逐年下降，草原植被盖度和森林覆盖率分别达到62%和30%。

——各方面体制机制更加健全完善。重点领域和关键环节改革取得决定性成果，鼓励创新的体制机制逐步完善。人民民主更加健全，司法公信力明显提高，民族团结、社会稳定、边疆安宁的政治局面进一步巩固。

各位代表，当前国内外环境正在发生深刻变化，我市发展既面临着严峻挑战，也面临着一系列新的重大机遇。国家加强供给侧结构性改革，为我市补齐短板、加快传统产业转型升级带来了新机遇；国家实施“一带一路”发展战略，自治区打造沿边经济带，深化与周边区域合作，为我市融入草原丝绸之路经济带、拓展发展空间带来了新机遇；国家深入实施西部大开发和振兴东北老工业基地战略，支持少数民族地区跨越发展，自治区加大东部盟市发展支持力度，为我市加快打造经济增长新引擎带来了新机遇。

今后五年，我们要努力推动经济持续健康发展，壮大综合经济实力。坚持发展第一要务，着力推进供给侧结构性改革，提高供给质量和水平。加快农牧业现代化，推进工业转型升级，发展现代服务业，保持经济中高速增长。推进一批打基础利长远的交通、水利、电力等重大基础设施项目，增强可持续发展后劲。

今后五年，我们要始终坚持创新发展理念，着力提高经济发展质量和效益。推进科技创新，提高区域创新、企业自主创新和科研成果转化能力。推进金融创新，提高金融服务实体经济能力。推进体制机制创新，深化重点领域改革。实施“互联网＋”行动计划，发展互联网经济，建设“智慧通辽”。

今后五年，我们要始终坚持协调发展理念，着力增强发展的整体性。推动城乡协调发展，健全城乡发展一体化体制机制。坚持以人为本、尊重自然、传承历史、绿色低碳理念，突出地域和民族特色，提高城镇规划建设管理水平。加强县域基础设施建设，做大做强县域特色优势产业。以“十个全覆盖”工程为抓手，建设美丽乡村。推进物质文明和精神文明协调发展，支持文化产业发展，基本建成覆盖城乡的公共文化服务体系。

今后五年，我们要始终坚持绿色发展理念，着力改善生态环境。牢固树立尊重自然、顺应自然、保护自然的生态文明理念，促进人与自然和谐共生。以主体功能区规划为基础，统筹各类空间规划。深入实施重大生态修复和建设工程，开展重点区域绿化，落实生态文明制度。加强环境综合整治，推动低碳循环发展，全面节约和高效利用资源。

今后五年，我们要始终坚持开放发展理念，着力提高开放型经济水平。积极对接国家“一带一路”发展战略，加强与俄蒙多领域合作，推进“锡赤通朝锦”中俄蒙国际海陆经济合作示范区建设。扩大同东北亚、欧美等国家和地区的经贸往来。深度融入东北经济区，主动接受京津冀、环渤海地区的辐射带动，积极承接发达地区产业转移。推进省域相邻地区交流合作，创新合作模式，打造区域经济新的增长极。

今后五年，我们要始终坚持共享发展理念，着力增进人民福祉。按照人人参与、人人尽力、人人享有的要求，坚守底线、突出重点、完善制度、引导预期，注重机会公平，保障基本民生，实现各族人民共同迈入全面小康社会。努力增加公共服务供给，大力实施脱贫攻坚工程，全面提升教育教学质量，千方百计提高城乡居民收入，建立更加公平更可持续的社会保障制度。

今后五年，我们要着力推进和谐通辽建设，营造安全稳定的发展环境。贯彻落实党的民族政策，促进各民族共同繁荣发展。加强法治政府建设，创新社会治理，统筹抓好信访维稳、安全生产、公共安全等领域工作，及时应对各类突发性事件，做好防灾减灾工作，建设平安通辽。

三、2016年主要工作

2016年是全面实施“十三五”规划的开局之年，是推进结构性改革的攻坚之年，是实施“十个全覆盖”工程的决胜之年，做好今年工作至关重要。市政府工作的总体要求是：全面贯彻中央、自治区和市委各项决策部署，紧紧围绕全面建成小康社会这个总目标，牢固树立和贯彻落实创新、协调、绿色、开放、共享发展理念，主动适应和引领经济发展新常态，坚持稳中求进工作总基调，统筹稳增长、促改革、调结构、惠民生、防风险各项工作，推动供给侧结构性改革，落实“宏观政策要稳、产业政策要准、微观政策要活、改革政策要实、社会政策要托底”五大政策和“去产能、去库存、去杠杆、降成本、补短板”五大任务，着力推动转型升级，着力培育新的发展动能，着力增强持续发展动力，着力保障和改善民生，努力实现“十三五”时期经济社会发展的良好开局。

主要预期目标是：地区生产总值增长8%，一般公共预算收入增长6.5%左右，限额以上固定资产投资增长13%，社会消费品零售总额增长9%，城乡常住居民人均可支配收入分别增长9%和10%。实现上述目标，重点做好以下工作。

（一）坚定不移推进结构性改革

全面落实“去产能、去库存、去杠杆、降成本、补短板”五大任务，以结构性改革推动经济提质增效。

积极化解过剩产能。我市去产能的主要任务是消化产能、转化利用产能，以消化产能倒逼产业转型升级，切实把我市的铝后加工和玉米加工等优势产业做成优势产能。铝后加工产业方面，坚定不移推动煤电铝一体化发展，以发电消化煤炭产能，以发展铝产业消化电力产能，以生产铝新材料消化原铝产能，实现循环发展，推动产能优势转化为高端产品优势。今年，争取启动国电投扎哈淖尔铝业二期38万吨电解铝项目建设，推进奇瑞集团1000万只铝轮毂制造、忠大铝业高铁车厢和汽车用铝板带、锦联二期100万吨和联晟35万吨铝板带箔、创源80万吨高强高韧铝合金及45万吨碳素等项目建设。铝及铝后加工产能分别达到295万吨和300万吨，新增铝新材料产品100个。玉米生物科技产业方面，推进梅花生物氨基酸产品生产线GMP改造、开封药业1000吨强力霉素等项目建设，新增玉米生物产品50个，通过开发新产品、打造新链条，实现玉米及淀粉产能就地转化。

扶持企业降低成本。开展降本增效行动，综合施策、多管齐下，打出一套务实管用的政策“组合拳”。争取大用户直供、扩大交易范围和交易电量，加快推进区域微型电网建设，降低企业用电成本；引进建设一批农资生产企业、发展节水高产高效农业、推进粮改饲和规模化养殖，降低农牧业生产成本；推广“铝水直供”模式，探索将玉米加工企业仓容纳入国家临储粮和地储粮行列等措施，降低企业中间成本；扩大“助保贷”融资规模，降低企业融资成本；清理涉企不合理收费项目，全面落实税费优惠政策，降低企业税费成本；完善企业运输“联络员制度”，实行“绿色通道”24小时畅通，降低企业物流成本；加大简政放权力度，清理规范中介服务，降低企业交易成本。努力让企业有效益、产品有市场、职工有收入、政府有税收。

消化房地产库存。加快进城农牧民市民化步伐，加大城镇棚户区改造货币化安置力度，实施存量商品房转公租房或回迁房政策，扩大住房需求。鼓励房地产企业兼并重组，盘活房地产库存积压的大量资金和资源，扶持开发企业与文化、旅游、体育、科教、养老产业融合跨界发展，培育房地产新业态，促进房地产市场持续健康发展。

扩大有效供给补齐短板。抓住当前原材料和投资品价格较低的有利时机，在城乡基础设施、农村牧区基本公共服务体系、现代产业体系建设等方面加大政策扶持和资金投入力度，加快补齐历史欠账，提高投资的有效性和精准性。

防范和化解金融风险。充分发挥金融良性杠杆作用，及时有效防控不良杠杆。积极引进和组建金融机构，发展多层次的资本市场，持续深化金融改革，推动金融部门不断扩大信贷投放规模，增强对实体经济的支撑保障能力。做好地方政府存量债务置换工作，完善全口径政府债务管理。加强风险监测预警，坚决守住不发生局部风险的底线。

（二）全力以赴稳定经济增长

保持经济中高速增长，必须发挥投资的关键作用，依靠转型升级和创新驱动，提升发展质量和效益，确保经济持续健康发展。

加快重点项目建设。着力扩大有效投资，限额以上固定资产投资达到1400亿元以上。产业方面，围绕“六大产业基地”建设，重点抓好165个续建项目、75个新建项目、60个拟引进项目、120个拟投产项目，增强可持续发展能力。今年力争实施500万元以上工业重点项目300个，完成工业固定资产投资677亿元。交通方面，加快通辽至京沈高铁连接线建设，完成通辽至霍林河、通辽至大虎山、通辽至四平等电气化铁路改造。加快建设通辽至鲁北高速公路，开工建设奈曼至营口高速公路、舍伯图至哈根庙一级公路、鲁北至开鲁二级公路，确保霍林河至阿力得尔一级公路、舍伯图至大兴二级公路、主城区北外环一级公路连接线建成通车。规划建设连通中南部旗县区的环线公路。启动通辽机场飞行区扩建工程，霍林郭勒机场通航运营。重大水利工程方面，推进“引绰济辽”、辽西北供水通辽支线、“引嫩济霍”等水利工程前期工作，开工建设嘎海山水库，完成西辽河治理二期工程。能源电力建设方面，推进北部区域微型电网改革试点工作，确保扎鲁特至山东青州±800千伏直流特高压电力外送通道开工建设，锦联二期2×66万千瓦火电机组项目建成投产。加快推进珠日河200万千瓦风电基地、科尔沁至新民500千伏输电线路、鸿骏铝电扎铝二期2×35万千瓦机组等项目前期工作。统筹抓好社会事业、民生领域和生态建设等方面的投资。拓宽投融资渠道，积极争取中央和自治区投资，推广政府与社会资本合作模式，扩大信贷和直接融资规模，保持足够的投资强度。

培育壮大优势主导产业。按照传统产业新型化、新兴产业规模化、支柱产业多元化的要求，紧紧盯住铝新材料、玉米生物科技、农畜产品加工产业，在壮大产业规模、提升产业层次、延伸产业链条上下功夫，再造主导产业发展新优势。大力扶持霍煤集团、锦联铝材、忠大铝业、联晟轻合金等铝后加工企业做大做强，促进铝新材料产业向新型化发展；大力扶持梅花生物、利牛生化、玉王科技、黄河龙生物、华曙生物等玉米深加工企业做精做细，促进玉米生物科技产业向高端化发展；大力扶持科尔沁牛业、邦杰肉牛、谷润肉业、牧合家肉牛、金锣集团、谷道粮原等企业做优做强，促进绿色农畜产品加工产业向终端产品延伸。增强康臣药业、蒙药股份、葛洲坝塔筒、金煤化工、福耀玻璃、欣意线缆、伯恩露笑等企业的市场竞争力，不断拓展产业发展新空间，构建多元发展、多极支撑的产业体系。

大力发展现代服务业。突出发展现代物流业。优化物流业空间布局，整合优化物流园区内部经营业态。完善提升以内陆港、保税物流中心为重点的开发区综合物流园区，以传化物流智能公路港、五洲城为重点的科尔沁现代物流园区，以快件中心、公路甩挂运输为重点的空港物流园区，以大连港公铁海多式联运为重点的木里图工业物流园区，以仓储加工、电子交易为重点的内蒙古东部煤炭交易中心等五大物流平台。培育大宗物流、冷链物流、快递物流、国际物流、电子商务物流及配送体系，加快建设东北区域物流中心。着力发展文化旅游产业。围绕培育“东北看草原、自驾游通辽”品牌，实施全域旅游发展战略，制定出台扶持文化旅游发展的政策措施，依托我市沙地、湿地、山地、草地等科尔沁草原自然风光，充分发挥我市民歌、版画、安代、乌力格尔等独特的科尔沁文化旅游资源，紧盯东北、华北区域性消费大市场，加快建设科尔沁500公里文化旅游风景大道自驾游服务系统，抓好阿古拉、莫力庙、扎旗山地草原等营地，舍伯图、图布信等旅游小镇，珠日河、可汗山等服务驿站建设，加快孝庄园、僧格林沁王府、大青沟、宝古图、银沙湾、额仑草原等沿线高A级景区景点建设。积极整合旅游节庆活动，组织开展2016年“科尔沁文化旅游季”活动，深入挖掘科尔沁马文化品牌，办好一年一度的“哲里木8·18赛马节”。培育市场文化主体，引导社会资本进入文化产业，推动文化创意等特色中小微文化企业发展，支持文化产业发展壮大。打造区域性、标志性的科尔沁文化品牌，促进文化旅游业全面融合发展。支持发展新型服务业。积极发展电子商务，完善通辽电子商务产业园功能，加快建设县域电子商务产业园及运营中心。搭建家庭服务业职业培训和公益性信息平台，完善支持生活性服务业发展政策体系，培育家庭服务、养老服务、健康医疗、体育休闲等一批标准化、品牌化、社会化的服务示范企业，满足多元化消费需求。

实施创新驱动发展战略。发挥科技创新的引领作用，建设通辽国家农业科技园区、铝新材料院士专家工作站等创新平台。开展铝新材料、玉米生物、蒙医药产业、大数据应用等重点科技攻关。以科技大市场、蒙药工程研究院、京蒙干细胞库项目为依托，创建自治区级高新技术产业开发区。加大非公经济扶持力度，完善非公经济阳光服务大厅功能，建设孵化平台，推动大众创业、万众创新。实施人才强市战略，创新人才引进培养和使用机制，让创新创富的活力充分涌流，为打造升级版新通辽蓄积持久动力。

发展壮大县域经济。各旗县市区要以工业园区为载体，着力壮大产业规模，强化产业配套，加快培育形成特色产业集群；以建设绿色农畜产品生产加工输出基地为目标，大力发展各具特色、优势明显、竞争力强的特色农牧业产业；以培育壮大农村牧区服务业为突破口，大力发展农村电商、乡村旅游、科技服务和信息服务等新兴服务业；以“十个全覆盖”和脱贫攻坚为抓手，全面促进城乡统筹发展；以“扩权强县”试点为契机，进一步深化农村牧区改革。市政府要在产业引导基金、简政放权、转移支付等方面给予旗县市区更大的倾斜。努力走出一条城乡统筹、产城融合、各具特色的县域经济可持续发展之路。

（三）提高农村牧区发展水平

深入实施农村牧区“十个全覆盖”工程。今年，全市筹措180亿元，完成454个行政嘎查村、1161个自然村和227个农牧场分场“十个全覆盖”工程，对2014年、2015年已经实施的1206个行政嘎查村、11个自然村、11个农牧场分场进行完善提高。实施农村牧区电网改造升级工程，提高供电能力，满足农牧民用电需求。开展“建美丽乡村，做文明主人”主题活动，让广大农牧民成为美丽家乡的建设者和维护者。

打好脱贫攻坚战。按照扶持对象精准、项目安排精准、资金使用精准、措施到户精准、因村派人精准、脱贫成效精准的要求，落实规划、项目、干部“三到村三到户”精准扶贫、精准脱贫举措。对435个贫困嘎查村逐村制定详细脱贫规划，对1.06万贫困户逐户制定精准脱贫方案，对4万贫困人口落实具体扶持措施。抓好5000名包扶干部驻村帮扶工作，实现扶贫规划、扶贫项目与包联干部无缝对接。确保年内通过发展特色产业减贫3.1万人，生态补偿减贫690人，易地扶贫搬迁减贫3230人，教育扶贫减贫2920人，社会保障兜底减贫2160人，确保“五个一批”扶贫举措落实到位，实现精准脱贫。

大力发展现代农牧业。必须坚定不移转方式、调结构、强基础，走产出高效、产品安全、资源节约、环境友好的现代农牧业之路。深入实施藏粮于地、藏粮于技战略。实施耕地质量保护与提升行动计划，推行玉米从播到收全程标准化，推广良种良法实用技术，新增节水高产高效粮食功能区30万亩、达到880万亩，巩固提升科尔沁区、科左中旗、开鲁县集中连片农业节水核心示范区。加快调整农牧业结构。面对资源环境约束、生产成本上升、主要农产品价格下降的形势，在稳定粮食产量的基础上，加快转变玉米“一粮独大”的种植结构，积极发展中药材、杂粮杂豆、红干椒、荞麦等特色种植业。积极推进粮改饲，引导农牧民种植饲料玉米达到450万亩，推进种养结合，为养而种、为牧而农，发展绿色循环农牧业。稳步提高畜牧业比重，抓好优质母牛繁育，实施母牛扩繁示范工程，新增肉牛存栏18万头，达到320万头，建设全国肉牛大市。推进农牧业标准化、产业化、品牌化。实施农牧业标准化生产、农畜产品产地证明和质量追溯，健全从农田牧场到餐桌的农畜产品质量全过程监管体系，切实保障农畜产品质量安全，培育“安全食品科尔沁”品牌，推动我市绿色农畜产品走向全国。完善龙头企业与农牧民利益联结机制，加大对领军龙头企业的扶持力度，拉紧拉长农牧业产业链，创新完善农畜产品流通体系，延伸终端消费产品供应链，促进三次产业融合发展。着力提高农牧民收入。培养新型农牧民，增强农牧民自我增收能力。拓宽农牧民增收渠道，引导农牧民发展规模种养业增加经营性收入，通过进城务工和经商创业增加非农收入，通过完善惠农惠牧政策、深化产权制度改革增加转移性收入和财产性收入，保持农牧民增收的持续性和稳定性。

（四）推进以人为核心的新型城镇化

认真落实中央、自治区和全市城市工作会议精神，按照“五个统筹”的要求，全面推进城镇工作。

推进“多规合一”，统筹城镇发展空间布局。规划科学是最大效益，规划失误是最大浪费，规划折腾是最大忌讳。统筹各类空间规划布局，按照不同主体功能区定位，增强国民经济和社会发展规划、生态环境保护规划、土地利用规划、城乡规划的协调性、一致性，推进“多规合一”。全面做好城市控制性详细规划，加快编制主城区近期建设规划，对城市重要地段和区域进行城市设计，加快编制市政基础设施和公共服务设施等专项规划，实现主城区规划全覆盖。旗县所有建制镇都要开展规划编制工作，全面完成31个自治区级重点镇规划编制工作。

实施民生城建，加快完善城镇功能。实施一批补短板、惠民生的市政项目，完善城市功能，提升城市品位。完善路网结构，完成霍林河大街东出口、建国北路、胜利北路等道路工程，开工建设新世纪大桥引道及南北立交桥、红光大桥等桥梁工程，改造提升和平路等既有道路及附属设施，对霍林河大街、创业大道等重点街路进行综合整治。加快市政管网建设，实施主城区新工二路、通辽铁路枢纽站等路段地下综合管廊工程，改造铁南片区和建国片区供热管网，翻建疏通污水和雨水管网。加快城镇公共服务设施建设，加强城镇环卫保洁、垃圾无害化处理、给排水、公共交通等市政公用基础设施建设。调整优化主城区商业布局，引进新型业态，培育专业市场集群。老城区重点发展百货大楼、万达广场、五洲城、长春欧亚等多极核心商圈，改造提升通辽批发城、团结路等专业批发市场；新城区加快建设万力城、红星美凯龙等大型商贸中心，建设仓储式购物中心。围绕完善功能、改善面貌和美化绿化，全面提升城关镇和重点镇建设管理水平，开展城镇综合整治和城乡清洁行动。结合农村牧区“十个全覆盖”工程，促进城乡基础设施互联互通、共建共享，缩小城乡基本公共服务供给差距。

推进智慧城市建设，提升城镇服务管理水平。深化城市管理体制改革，明确市区两级管理范围和管理主体，制定管理责任清单。推进城市管理综合执法体制改革，加快城市管理立法。落实智慧城市总体规划，争取智慧城市国家试点，加快建设“智慧通辽”，重点实施智慧公交、智慧教育、智慧医疗、智慧食品药品监管、智慧旅游等工程，实现信息共享和互联互通，促进城市管理向网格化、智能化、人性化方向发展。

扎实开展“双城同创”工作，建设文明宜居城镇。加大全国文明城市和国家卫生城市创建工作力度，营造浓厚创城氛围，细化工作任务，强化督查指导，落实测评体系和创建标准，从群众反映最强烈的私搭乱建、占道经营、物业管理、环境污染、交通秩序、回迁安置等问题入手，开展重点难点集中攻坚。对“双城同创”的好经验好做法，我们要坚持不懈抓下去，决不搞花架子、不搞一阵风，不断积聚城市发展正能量。加强城市绿线管理，有计划回购闲置土地建设游园绿地。实施辽河公园提升扩建工程，建设园内及辽河南堤慢行系统，争创中国人居环境范例奖。完成西拉木伦公园、人民公园提升改造工程。全面启动各旗县城关镇创建自治区园林县城活动。

（五）加强生态文明建设

突出抓好生态修复。保持良好生态环境，做到不欠新账、多还旧账。继续实施重大生态修复工程，完成科尔沁沙地“双千万亩”综合治理346万亩。加快建设罕山、大青沟、乌斯吐、老哈河南岸沙带等生物多样性保护示范区和生态科技示范园，启动建设科左后旗乌旦塔拉生物多样性示范区。大规模推进国土绿化行动，全面提高市内干线公路绿化水平，完成1081个嘎查村村屯绿化，创建国家森林城市、国家生态园林城市和绿化模范城市。

加大环境治理力度。完善污染物总量控制制度，集中治理重点流域、工业园区水污染。加强水源地周边环境整治和保护。深入推进节能减排工作，促进工业污染源达标排放，加大环境监测监管力度，有效防控环境风险。完善城镇污水收集处理系统，推动城镇生活污水、工业园区污水及垃圾处理设施全覆盖并稳定运行。开展烟尘治理，防治大气污染。加大“三滥”治理力度，坚决打击滥开荒、滥樵采、滥放牧行为。

落实生态文明制度。划定农牧业生产空间和生态空间保护红线，落实最严格的水资源管理制度，完成地下水超采区边界划定，加强用水定额和计划管理。落实最严格的耕地保护制度。完善沙地沙漠、矿产资源开发保护管理和生态修复等制度。建立科学合理的空间治理体系，严格落实用途管制、领导干部自然资源资产离任审计、差异化绩效考核等生态文明制度。

（六）全面深化改革扩大开放

加大改革攻坚力度。坚持聚焦大事、突出重点，统筹推进经济生态及社会事业领域改革工作。深化行政管理体制改革。推行市旗两级网上审批、并联审批，对保留的行政审批事项进行流程再造，提高审批服务效率。完善权力清单，理出责任清单。按要求完成公务用车改革。深化商事制度改革。推行电子营业执照，实现全程电子化登记管理和服务。深化电力市场改革。扎实推进北部区域微型电网和输配电价改革试点，增加大用户直供交易电量。深化国有企业改革。鼓励民间资本参与国有企业改革，发展混合所有制经济。抓好霍煤集团等国有企业改制重组，盘活国有资产，实现国有资产保值增值。深化农村牧区综合改革。农村牧区土地和草牧场确权登记颁证试点工作通过综合验收，完成农村牧区集体土地所有权和使用权登记发证工作。推进土地、林业、水利、供销社、农村牧区金融改革。深化食品药品监管体制改革。建立统一权威的食品药品监管体系，筑牢食品药品安全监管“网底”。

实施更加主动的对外开放战略。加快融入“一带一路”发展战略，加强与俄蒙经贸人文合作。大力发展陆港经济、空港经济，加快建设通辽保税物流中心和锦联、霍煤鸿骏公用型保税仓库。深度融入东北经济区一体化发展，主动接受京津冀、环渤海地区的辐射带动，通过精准招商实现产业集群式发展。年内力争引进亿元以上项目100个，到位资金600亿元以上。

（七）提高民生和社会事业发展水平

做好民生重点工作。在全面落实自治区低收入农牧民家庭1吨“暖心煤”、低保家庭大学生每年1万元助学金、零就业家庭1人稳定就业等民生政策的基础上，重点做好十件民生实事。实施百姓安居工程。改造农村牧区危房7.14万户，改造城镇棚户区1.2万户。全市既有建筑节能改造完成200万平方米。主城区改造老旧住宅小区45个，改造老化管网20条，对老城区58栋“土楼”进行集中供热改造，实现基本物业服务全覆盖。实施城市服务管理提升工程。主城区新改造建设50座免费公共厕所。增加100辆新能源公交车。新建20处停车场。增加公共自行车布点50处，投放自行车总数达到2000辆。实施供水改造和水质提升工程。完成农村牧区饮水安全工程660处，解决43.3万人饮水安全问题。完成主城区35公里供水主管网和54个老旧小区供水管网改造，完成第二水厂、第三水厂除铁除锰工艺改造和26个二次加压水箱改造，居民饮用水检测能力由42项提升到106项，达到省会城市标准。实施大气污染防治工程。集中整治燃煤小锅炉，逐步取缔主城区洗浴业、餐饮业和商业网点的燃煤炉灶，实行清洁能源替代改造。采取引导鼓励政策，提高秸秆转化率，有效控制秸秆焚烧，改善空气质量。实施养老服务建设工程。开工建设通辽市老年人养护中心，新建3所社会化养老服务机构。建设农村牧区互助养老幸福院80个，安置农村牧区低保、五保、重点优抚对象等特殊群体老年人3000人。实现“12349便民为老服务平台”全覆盖，在公共场所规划建设方便老年人和残疾人的无障碍设施。实施城乡安全畅通工程。在全面取消市内所有公路区间测速和车载流动测速的基础上，依法对境内高速公路适当提速，合理调整其他公路的限速规定。对城乡公路交通标识进行全面升级改造，抓好中心城区和库伦旗试点工作。实施食品安全监管工程。推进“食品药品安全通辽”建设，各旗县市区食品加工园区全部投入使用，全市改造食品加工小作坊500家，食品流通示范店达到4300家，餐饮示范店达到1000家。完成主城区1000家小餐饮店改造，投入流动小餐车500台。实施创业就业工程。提供就业岗位3万个，新增城镇就业1.7万人，农牧民劳动力转移就业45万人，开展就业创业培训3万人。落实蒙语授课高校毕业生就业政策，对初中毕业未升入高中学生实行免费职业培训。实施办学条件改善和学校布局优化工程。全面改善21所农村牧区蒙古族小学住宿条件，解决“大通铺”宿舍问题。推进教育信息化，加快“三通两平台”建设，主城区的教育信息化水平要得到明显提升。优化老城区学校布局，开工建设科尔沁区三中、六中和民族幼儿园，迁址新建铁路一小和铁路幼儿园；完善开发区学校布局，调整中小学和幼儿园建设规划，开发区实验小学建成使用。实施贫困人口免费体检工程。从今年开始，市政府负责筹集资金，各旗县市区统一组织，对贫困人口每年进行一次免费体检。在做好民生实事的基础上，我们还将加大社会保障和救助力度，提高城乡低保、农村牧区五保、城镇“三无”人员和孤儿供养标准及补助水平。稳步推进机关事业单位养老保险制度改革，落实城镇职工养老保险自治区统筹政策。规范市级医疗保险统筹，实现异地就医结算。加大城乡医疗救助力度，提高临时救助水平。

提升社会事业发展水平。优先发展教育事业。加快推进教育领域综合改革，推进义务教育均衡发展，规范办学行为，完善高中阶段教育招生制度。优先发展民族教育，打造通辽民族教育品牌。突出抓好职业教育，加大职业教育资源整合力度，促进高中阶段教育协调发展。推进健康通辽建设。全面深化医药卫生体制综合改革，加快医疗卫生计生信息化区域平台建设，完善基层公共卫生服务体系。支持中心卫生院建设，实现行政嘎查村标准化卫生室全覆盖，为试点嘎查村卫生室配备多功能健康一体机。参合农牧民政府补助标准和个人筹资标准分别提高到每人每年420元、120元。强化蒙中医药服务能力建设，巩固提高全国蒙中医先进市创建成果。做好艾滋病等重大疾病防控。落实全面两孩政策，加强妇幼保健能力建设，全面提高计划生育服务管理水平。繁荣发展文化体育事业。制定出台相关扶持政策，保护和传承好民族文化，实施文化精品工程，打造科尔沁文化品牌。完善公共文化服务体系，加快僧格林沁王府、市图书馆新馆建设，实施市博物馆改造工程。广泛开展全民健身活动，深化足球改革，大力发展校园足球，建成50片笼式足球场地，启动建设自治区级足球青年训练中心。全面提升各项社会事业发展水平，为社会全面进步作出新贡献。

维护社会和谐稳定。开展民族团结进步活动，创建全国民族团结进步示范市。依法管理宗教事务，维护宗教和睦、社会和谐。全面推进警务共同体建设，完善立体化治安防控体系，确保社会治安秩序持续稳定，提高“平安通辽”建设水平。加强安全生产工作，落实安全生产责任制，强化矿业、消防、危爆品、道路交通等重点行业领域安全监管，及时排查治理安全隐患，防止重特大事故发生。完善防灾减灾和应急救援体系，提高灾害预防和救助能力。启动“七五”普法。推进信访法治化，保障群众合理合法诉求通过法治渠道得到解决。加强国防动员和后备力量建设，推动军民深度融合发展。褒扬家庭美德，注重家教家风，促进家庭和睦，使千千万万个家庭成为社会和谐的重要基点。

（八）提升政府执行力和公信力

依法全面履行政府职能。用法治思维和法治方式履行职责，切实做到“法定职责必须为，法无授权不可为”。健全依法决策机制，严格执行重大行政决策法定程序。规范公正文明执法，加强政府法制和法律顾问队伍建设。依法接受人大法律监督，自觉接受政协民主监督，主动接受媒体和公众监督，让政府工作在法治轨道上运行。

切实提高执行落实能力。“为官避事平生耻”。要切实解决“想不想干、能不能干、干什么、抓紧干”的问题，真正做到守土有责、守土负责、守土尽责。大力解放思想，坚持变中求进、变中突破，更好地引领经济发展新常态。坚持以严的要求和实的作风，主动作为，勇于担当，确保政府推进的每项工作都能落到实处，政府承诺的每件实事都能得到兑现。

提升政府公共服务水平。各级政府要坚持问题导向，简化优化公共服务流程，方便群众办事创业，全面梳理并公布公共服务事项目录，编制办事指南，取消无法律依据的证明和手续，打通服务群众“最后一公里”。

增强拒腐防变能力。始终把政治纪律和政治规矩挺在前面。完善惩治和预防腐败制度，强化对行政权力的制约和监督，加强行政监察和审计监督，推动政府系统党风廉政建设。市政府工作人员特别是党员干部要带头贯彻《中国共产党廉洁自律准则》和《中国共产党纪律处分条例》，守住纪律“底线”。

各位代表，明年我们将迎来自治区成立70周年，这是通辽各族人民的一件大事。我们一定要以高度的政治责任感和历史使命感，把迎庆工作与经济社会发展紧密结合起来，通过一个个工程的实施、一项项任务的完成、一件件实事的落实，以人民群众满意的工作实绩向自治区成立70周年献礼！

各位代表，“十三五”蓝图已经绘就。时代赋予使命，奋斗创造未来。让我们在市委的坚强领导下，紧紧依靠全市各族干部群众，守望相助、锐意进取、真抓实干，全力推动我市经济社会持续稳定健康发展，为把祖国北部边疆这道风景线打造得更加亮丽而努力奋斗！
